# Associations of the circulating levels of cytokines with the risk of myeloproliferative neoplasms: a bidirectional mendelian-randomization study

**DOI:** 10.1186/s12885-024-12301-x

**Published:** 2024-04-26

**Authors:** Hao Xiong, Huitao Zhang, Jun Bai, Yanhong Li, Lijuan Li, Liansheng Zhang

**Affiliations:** 1 Department of Hematology, The Second Hospital of Lanzhou University, Lanzhou, China; 2 Department of Hematology, The Affiliated Hospital of Southwest Medical University, Luzhou, China

**Keywords:** Myeloproliferative neoplasms, Mendelian randomization, Inflammatory, Cytokines

## Abstract

**Objective:**

In the pathogenesis of myeloproliferative neoplasms (MPN), inflammation plays an important role. However, it is unclear whether there is a causal link between inflammation and MPNs. We used a bidirectional, two-sample Mendelian randomization (MR) approach to investigate the causal relationship between systemic inflammatory cytokines and myeloproliferative neoplasms.

**Methods:**

A genome-wide association study (GWAS) of 8293 European participants identified genetic instrumental variables for circulating cytokines and growth factors. Summary statistics of MPN were obtained from a GWAS including 1086 cases and 407,155 controls of European ancestry. The inverse-variance-weighted method was mainly used to compute odds ratios (OR) and 95% confidence intervals (Cl).

**Results:**

Our results showed that higher Interleukin-2 receptor, alpha subunit (*IL-2rα*) levels, and higher Interferon gamma-induced protein 10 (*IP-10*) levels were associated with an increased risk of MPN (OR = 1.36,95%CI = 1.03–1.81, *P* = 0.032; OR = 1.55,95%CI = 1.09–2.22, *P* = 0.015; respectively).In addition, Genetically predicted MPN promotes expression of the inflammatory cytokines interleukin-10 (*IL-10*) (BETA = 0.033, 95% CI = 0.003 ~ 0.064, *P* = 0.032) and monokine induced by interferon-gamma (*MIG*) (BETA = 0.052, 95% CI = 0.002–0.102, *P* = 0.043) and, on activation, normal T cells express and secrete *RANTES* (BETA = 0.055, 95% CI = 0.0090.1, *P* = 0.018).

**Conclusion:**

Our findings suggest that cytokines are essential to the pathophysiology of MPN. More research is required if these biomarkers can be used to prevent and treat MPN.

**Supplementary Information:**

The online version contains supplementary material available at 10.1186/s12885-024-12301-x.

## Introduction

The chronic hematological malignancies known as myeloproliferative neoplasms (MPN), which include polycythemia vera (PV), essential thrombocythemia (ET), and myelofibrosis (MF), advance at varying rates [1]. The incidence rates of PV, ET, and PMF are estimated to be 0.5 to 4.0, 1.1 to 2.0, and 0.3 to 2.0 per 100,000 people, respectively. It is reported that nearly 10–15% of patients with MPN progress to AML [2], more than 20% will develop thrombosis during the disease, and approximately 6.2% of newly diagnosed patients will suffer hemorrhage [3, 4]. The presence of these symptoms mentioned above raises the rate of disability and mortality in MPN patients [5] and imposes a huge economic burden on the family and society. The most common feature of MPN is hyperactivation of Janus kinase 2 (*JAK2*) signaling, which is caused by acquired mutations in *JAK2*, *MPL*, and *CALR* [6]. However, clinically used *JAK2* inhibitors such as Ruxolitinib and Fedratinib have limited efficacy, high toxicity, and are prone to drug resistance [6–8]. Therefore, increased awareness of the pathogenic components may offer clues for halting the disease's course and creating novel treatments.

The chronic inflammatory environment is one of the typical features of myeloproliferative neoplasms, where inflammation is tightly intertwined with tumor clones, providing a permissive micro-environment for disease progression [9–11]. Inflammatory cytokines are essential immune mediators in the physiology and disease process of MPN and not only play a significant role in inflammatory pathology but are also inextricably linked to the development of the disease [9, 12]. *GM-CSF*, *IL-1*, *IL-4*, *IL-5*, *IL-6*, *IL-10*, *IFN-2*, *MIP-1*, *IL-12*, and *TNF-α* were shown to have higher cytokine levels in treatment-naive patients in all three MPN groups when compared to age-matched control participants, according to an observational study [9]. In addition, serum *IL-2* and soluble IL-2 receptor alpha (*sIL-2rα*) increased as patients with MPNs progressed to advanced clinical stages [13], and serum *IL-2*, *sIL-2rα*, and *IL-6* levels were positively correlated with bone marrow neovascularization, indicating that increased inflammatory responses may be connected to the course of MPN disease [14], suggesting that MPN patients may benefit from using cytokines as a tool for illness monitoring [15]. However, little is known about the mechanisms and duration of inflammation in MPNs [16]. The origins of the increased cytokine production in MPNs (alterations, others?) and whether inflammation may occur before the development of *JAK2/CALR/MPL* gene mutations are still up for dispute. Observational studies are prone to common biases such as reverse causality and residual confounding [17] and have limitations such as small sample sizes and short follow-up periods. These studies, however, only addressed a small subset of inflammatory cytokines and did not take into account how other physical factors can affect changes in inflammatory cytokine levels. Determining whether variations in inflammatory cytokines cause the development of MPN or whether MPN development influences the microenvironment and causes variations in inflammatory cytokines is crucial. Investigating the precise nature of the connection between inflammatory cytokines and MPN is crucial from a therapeutic standpoint given the lack of knowledge regarding the etiology of MPN.

To establish a link between inflammatory cytokines and MPN, we applied Mendelian randomization (MR).MR has the advantage of reducing confounding variables and measurement error, as well as addressing the limitations of traditional observational studies mentioned above. This approach can effectively avoid bias caused by reverse causality [18]. The greatest level of evidence hierarchy outside of randomized controlled trials is provided by MR, which uses genetic variation as an instrumental variable (IV), which has been a dependable tool for getting reliable estimates of the causal influence of numerous risk variables on health [19]. In the current investigation, we used a two-sample MR design to methodically evaluate the potential causal link between inflammatory cytokines and MPN risk. Additionally, reverse MR analysis was done to determine how MPN affected cytokines.

## Methods

### Study design

The Mendelian randomization design method uses publicly available datasets from extensive genome-wide association studies (GWAS) for risk factors and disease to examine whether exposure has a causal effect on disease emergence. As a genetic instrumental variable analysis, MR Uses single nucleotide polymorphisms (SNPs) as instrumental variables for the risk factor of interest. SNP_S_ are randomly assigned at meiosis and are not subject to reverse causality bias, so the Mendelian randomization approach can overcome unmeasured confounders and lead to more reliable causal inferences [20].

To determine the relationship between inflammatory cytokine levels and the risk of MPN, we conducted a bidirectional Mendelian randomized trial. As the summary statistics from published research are made available to the public, the institutional review board did not need to approve our study's ethics further. In Supplementary Table 1, the features of the data used in this investigation are displayed.

### Data sources and instruments

#### Cytokine

The data on inflammatory cytokines was obtained from a meta-analysis published in 2017 that summarized data from genome-wide association studies (GWAS) carried out with three Finnish cohorts (YFS and FINRISK, 1997 and 2002), totaling 8,293 Finns. The original publication has information about the cytokine assays, inclusion standards, etc. [21]. In MR analysis, *P*-values are used to measure whether there is an association between genetic variants and exposure factors. We set the *P* value to find the genetic variant loci associated with the trait. The independence of the selected instrumental variables was further ensured by removing all SNPs with linkage disequilibrium (LD) to avoid biased results (parameters kb = 250, *r*^*2*^ = 0.001). Calculate the F-statistic [using the formula: *F* = (N-2)*R2/(1-R2), N is the sample size]to assess the extent of weak instrument bias, *F* > 10 suggests that full instrumental SNPs are sufficiently strong to lessen any potential bias, while an F-statistic ≤ 10 implies weak instruments [22].Initially, we used *P* < 5 × 10^–8^ as a criterion to look for instrumental factors, and we discovered that the majority of cytokines had either no SNPs or only a few SNPs [3]. To get as many cytokines as possible into the study. Furthermore, we chose IVs using a permissive significance criterion (*P* < 5 × 10^–6^). Further, we use the parameter kb = 250, *r*^*2*^ = 0.001 to eliminate the linkage disequilibrium among variables. Supplementary Tables 2 and 3 provide extensive details on the features of the IVs.

#### Myeloproliferative neoplasms

The MPN Patients' data were obtained from publicly available GWAS data, which we downloaded from open GWAS (https://gwas.mrcieu.ac.uk/). The data come from UK Biobank, which is a cohort study conducted between 2006 and 2010. The study collected in-depth genetic and phenotypic data on approximately 500,000 people across the United Kingdom. The data we downloaded for analysis included 1086 MPN patients and 407,155 controls [23]. The original article describes the criteria and procedures for the data's quality control [23]. Erythrocytosis, primary thrombocythemia, myelofibrosis, chronic myeloid leukemia, and chronic myeloproliferative illness are all included in the UKBB's description of the MPN phenotype. Those who had polycythemia vera, essential thrombocythemia, myelofibrosis, chronic myeloid leukemia, or malignant mastocytosis were additionally labeled as cases if they had self-reported cancer, self-reported sickness code, or histology of cancer tumor code. In the MPN dataset, we screened SNPs as instrumental variables according to the following criteria: (*P* < 5 × 10^–8^, *r*^*2*^ = 0.001, kb = 250 kb). Five SNPs were kept as separate MPN IVs. Inverse MR analyses were performed using these SNPs to examine the genetic influence of MPN on the amount of cytokines.

#### Bioinformatics analysis

We use the data set from the GEO database for gene expression spectrum analysis (https://www.ncbi.nlm.nih.gov/geo/query/acc.cgi). The GSE103237 dataset is based on the GPL13667 platform and consists of 26 polycythemia vera, 24 essential thrombocythemia, and 15 normal bone marrow samples. The GSE136335 dataset is based on the GPL17586 platform consisting of 8 myelofibrosis patients and 6 normal bone marrow samples. We used the "Ggpubr" package to create violin plots to visualize the expression of *IP-10*, and *IL-2ra* in the three subtypes of MPN. The diagnostic value of *IP-10* and *IL-2ra* was analyzed by the receiver operating characteristic (ROC) curve. The expression of genes was analyzed using the "pROC" package (v1.18.2), and genes with the area under the curve (AUC) > 0.7 were considered potential diagnostic markers.

#### Statistical analysis

We harmonized the effect of SNPs on inflammatory cytokines and MPNs before the Mendelian randomization analysis. Furthermore, we used the two-sample MR software package for MR analysis. The inverse variance weighted(IVW) methods were used to assess potential causality, while weighted median and MR-Egger regression methods were used as complementary methods for causality between inflammatory cytokines and MPN [24]. The MR-Egger method was used for multiple validity testing. We used Cochran's Q test to assess heterogeneity in IVW [25]. MR-PRESSO was used to detect the presence of outliers [26], and leave-one-out analyses were used to verify whether the causal effect depended on a single variant. We also performed instrumental strength tests using the F statistic, with *F* > 10 indicating sufficient strength [27]. Eventually, if the IVW method result is significant (*P* < 0.05), even if the findings of other techniques are not significant and there is no pleiotropy or heterogeneity discovered, it may be regarded as a good result, given that the beta values of the other methods are in the same direction [28].

Based on the number of cytokines, we used the Bonferroni approach to compensate for multiple comparisons and set statistical significance at a *P*-value < 1.22 × 10^−3^ (0.05/41) level. If a *P*-value was between 1.22 × 10^−3^ and 0.05, we considered suggestive evidence for a potential causal association. The majority of the work mentioned above was completed using the R analysis program (version 4.3.0), which was applied to the relevant R package, including Two-sample MR, data array, etc.

## Results

### Instrumental variables

**Fig. 1:**
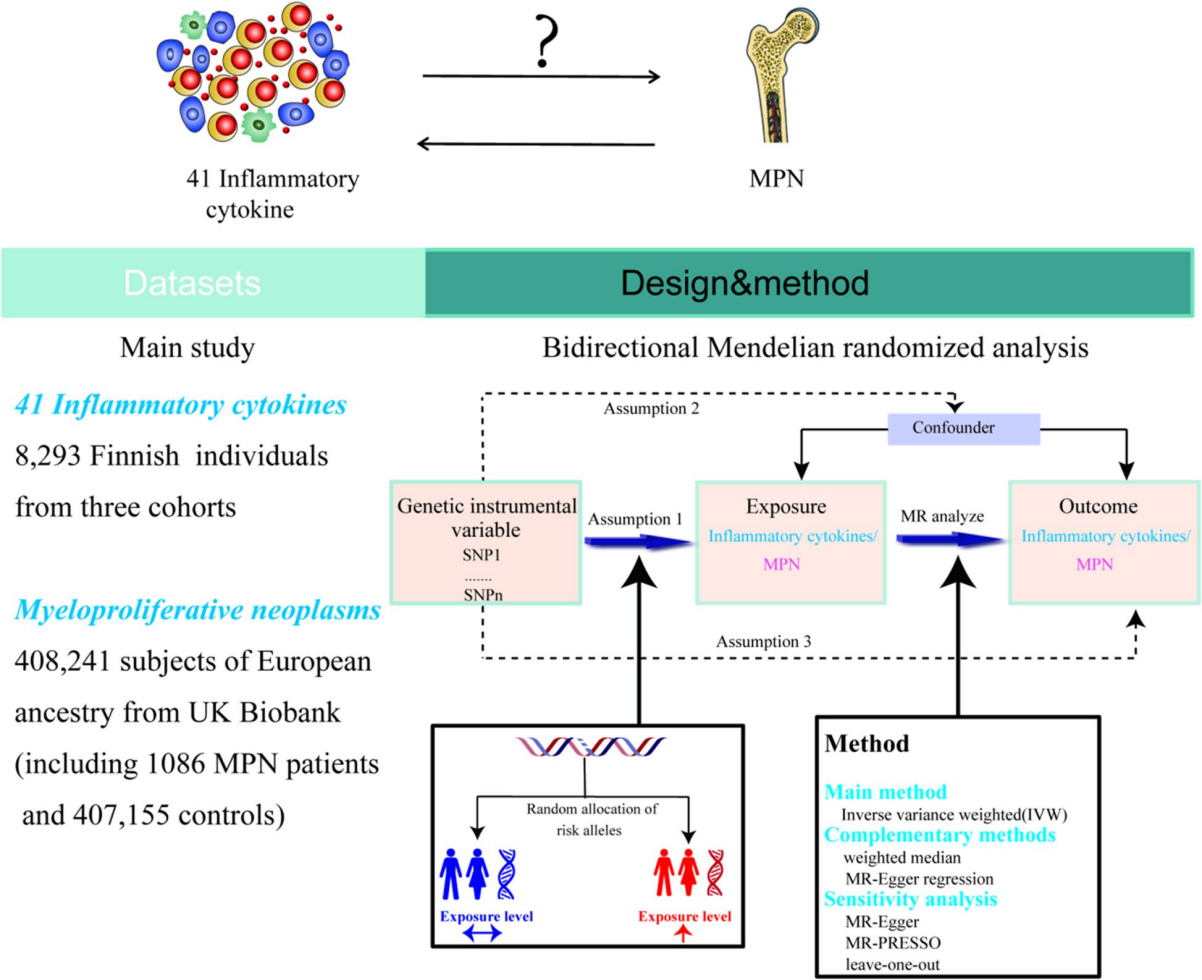
Study design overview and assumptions for MR Design. Assumption 1: IVs were not related to Confounders; Assumntion2: a strong correlation between iVs and exposure; Assumption 3: IVs can affect outcomes only through exposure and not through other pathways

Figure 1 offers a flowchart of the full-text logic. In the study of the effect of inflammatory factors on MPN, we screened instrumental variables according to the criteria of *P* < 5 × 10^–6^ and *r*^*2*^ < 0.001. A total of 354 SNPs linked to 41 cytokines were used for MR analysis after being harmonized with the outcome variable MPN (Supplementary Table 1).To analyze the effect of MPN on inflammatory factors, we used MPN as an exposure factor, By using such criteria (*P* < 5 × 10^–8^, *r*^*2*^ < 0.001), a total of five SNPs were obtained for subsequent analyses, and specific SNP information can be found in Supplementary Table 2. These SNPs' F statistics, which ranged from 20.77 to 781.85(Table 1), showed that the instrument was sufficiently reliable to rule out the possibility of a null relationship brought on by instrument bias.

**Table 1:** Details of the number of genetic instruments and F-statistic for each cytokine and growth factor

Exposure	Abbreviations	No. of SNPs	F-statistic	R2
Beta nerve growth factor	β-NGF	4	25.72(20.83–35.43)	0.029
Cutaneous T-cell attracting (CCL27)	CTACK	10	39.41(20.84–139.89)	0.097
Eotaxin (CCL11)	Eotaxin	18	47.81(20.85–203.72)	0.095
Basic fibroblast growth factor	FGF-basic	8	24.77(20.79–25.40)	0.024
Granulocyte colony-stimulating factor	G-CSF	5	25.37(21.85–25.80)	0.016
Growth regulated oncogene-α (CXCL1)	GRO-a	7	100.62(21.17–248.86)	0.166
Hepatocyte growth factor	HGF	7	30.53(21.23–55.42)	0.025
Interferon-gamma	IFN-γ	8	23.69(21.47–24.99)	0.024
Interleukin-1 receptor antagonist	IL-1rα	6	22.84(20.89–24.25)	0.036
Interleukin-1-beta	IL-1β	4	22.81(21.78–23.04)	0.027
Interleukin-2	IL-2	7	23.86(20.94–27.62)	0.045
Interleukin-2 receptor, alpha subunit	IL-2rα	6	49.28(21.12–170.04)	0.074
Interleukin-4	IL-4	11	26.80(21.22–28.03)	0.035
Interleukin-5	IL-5	7	25.23(21.19–38.39)	0.049
Interleukin-6	IL-6	8	23.87(21.23–30.71)	0.023
Interleukin-7	IL-7	4	60.73(20.84–169.54)	0.066
Interleukin-8 (CXCL8)	IL-8	5	22.77(21.10–23.85)	0.031
Interleukin-9	IL-9	4	23.50(21.54–26.37)	0.025
Interleukin-10	IL-10	14	46.17(21.26–300.09)	0.077
Interleukin-12p70	IL-12p70	13	76.58(20.89–559.19)	0.100
Interleukin-13	IL-13	9	56.52(20.88–292.36)	0.124
Interleukin-16	IL-16	8	44.72(21.99–133.68)	0.092
Interleukin-17	IL-17	6	25.45(20.95–35.94)	0.019
Interleukin-18	IL-18	11	48.72(21.83–100.11)	0.128
Interferon gamma-induced protein 10 (CXCL10)	IP-10	7	24.20(21.24–26.86)	0.044
Monocyte chemotactic protein-1 (CCL2)	MCP-1	18	50.77(21.45–201.44)	0.099
Monocyte-specific chemokine 3 (CCL7)	MCP-3	6	25.08(20.77–26.39)	0.121
Macrophage colony-stimulating factor	M-CSF	5	24.30(20.96–30.98)	0.046
Macrophage migration inhibitory factor (glycosylation-inhibiting factor)	MIF	8	26.56(21.25–38.68)	0.057
Monokine induced by interferon-gamma (CXCL9)	MIG	7	27.44 (21.62–41.56)	0.049
Macrophage inflammatory protein-1α (CCL3)	MIP-1α	2	22.24(21.83–22.40)	0.012
Macrophage inflammatory protein-1β (CCL4)	MIP-1b	28	120.68(21.55–603.64)	0.290
Platelet-derived growth factor-BB	PDGF-bb	12	52.83(21.17–240.19)	0.071
Regulated on activation, normal T Cell expressed and secreted (CCL5)	RANTES	9	26.21(20.84–34.93)	0.064
Stem cell factor	SCF	9	28.01(22.13–48.08)	0.029
Stem cell growth factor beta	SCGF-β	15	95.48(20.90–96.22)	0.147
Stromal cell-derived factor-1 alpha (CXCL12)	SDF-1α	5	22.42(20.91–23.36)	0.014
Tumor necrosis factor-alpha	TNF-α	2	22.88(22.40–23.07)	0.013
Tumor necrosis factor-beta	TNF-β	3	23.35(21.8–24.60)	0.043
TNF-related apoptosis-inducing ligand	TRAIL	13	42.63(21.28–138.48)	0.063
Vascular endothelial growth factor	VEGF	15	90.67(20.88–781.85)	0.160
Myeloproliferative neoplasms	MPN	5	64.80(45.07–117.72)	0.001

### Effect of inflammatory cytokines on MPN

**Fig. 2:**
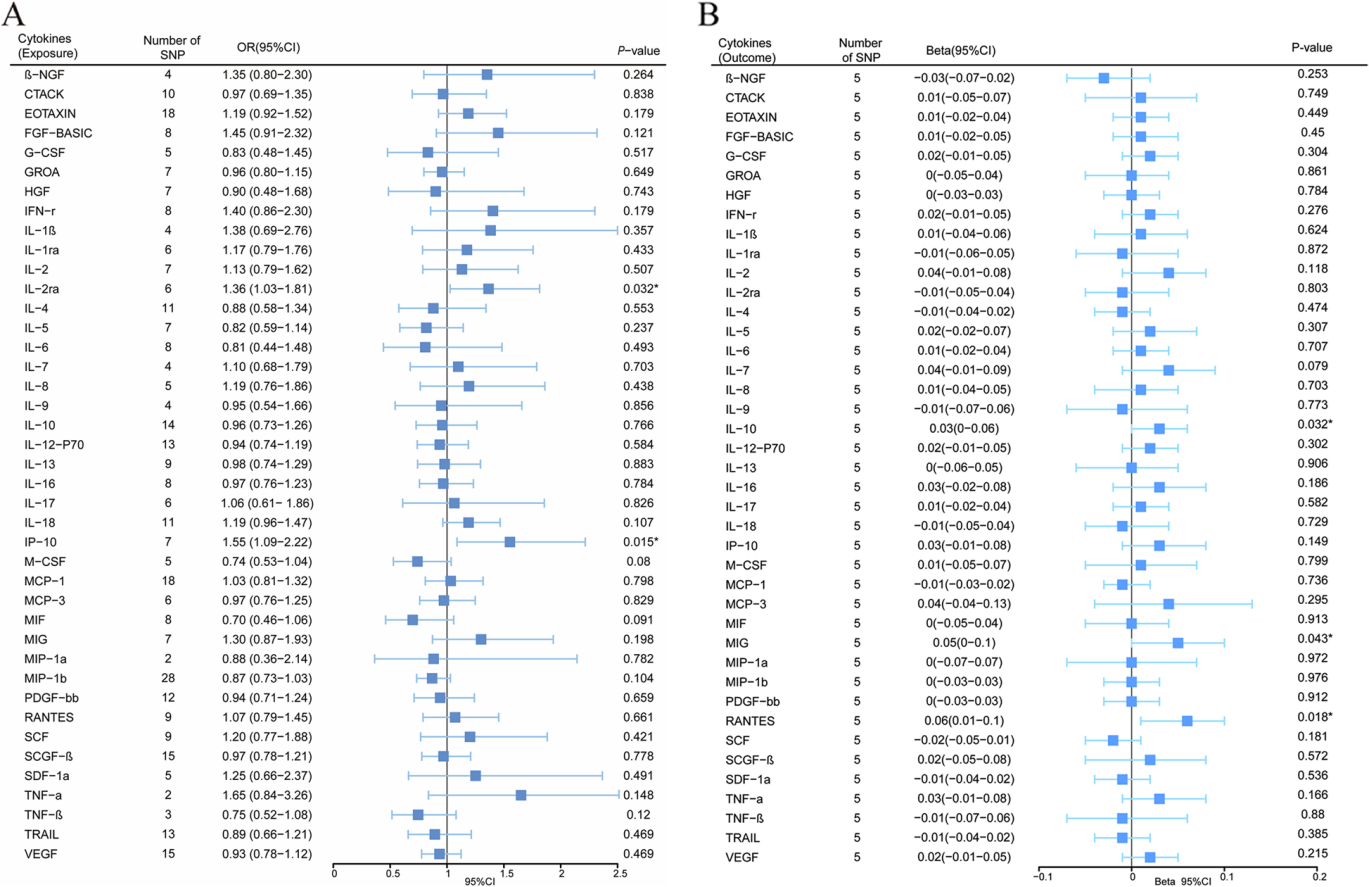
Forest plot of Mendelian randomization analysis of the correlation between inflammatory cytokines and risk of myeloproliferative neoplasms (MR analysis method using IVW). **A**: Effects of 41 inflammatory cytokines on myeloproliferative tumors. **B**:Effects of myeloproliferative neoplasms on 41 inflammatory cytokines(*P < 0.05)

**Fig. 3:**
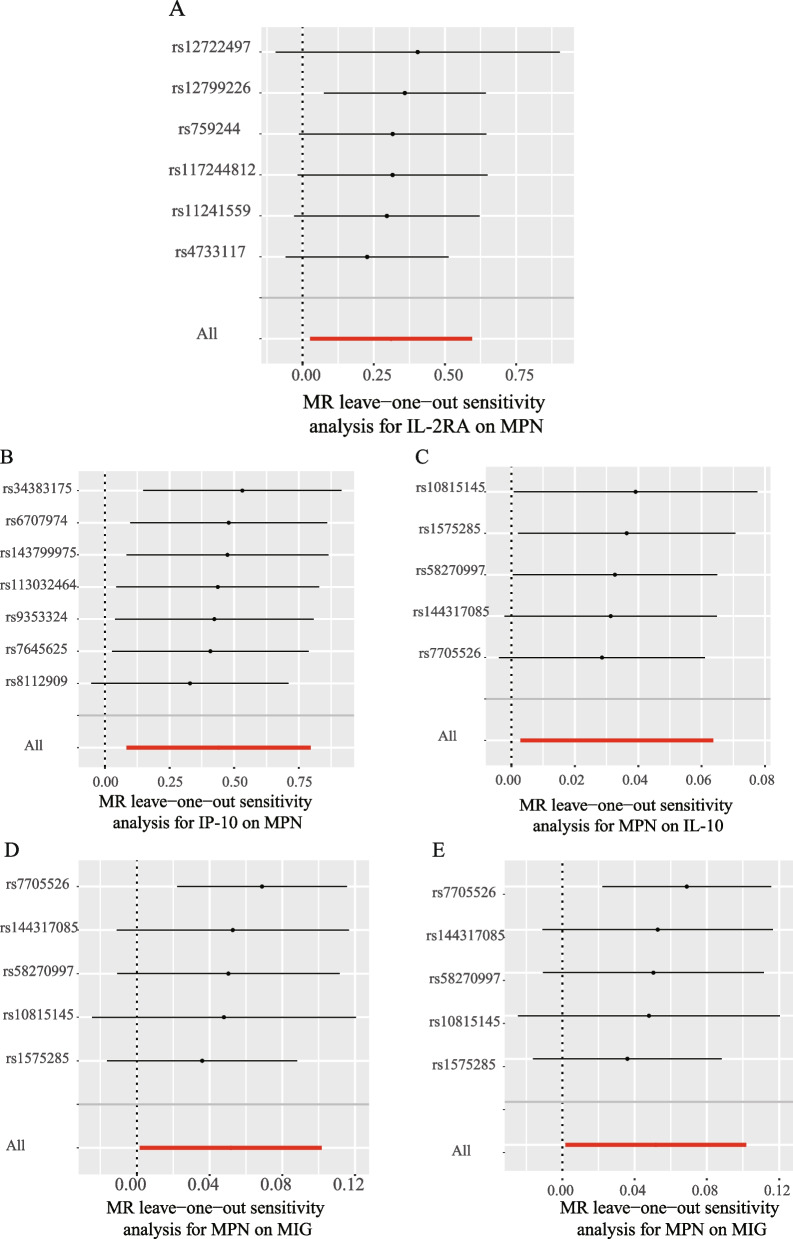
Leave-one-out analysis of bidirectional mendelian randomization in cytokines and MPN.The left side of the forest plot represents the SNPs for which the leave-one-out analysis was performed, and the short line parallel to the x-axis represents the 95% confidence interval for the OR/beta value of the MR analysis after excluding the corresponding SNPs. As shown in the figure, the overall error line does not change much after excluding each SNP, and all OR/beta values are on the 0 side, indicating that the results are reliable (**A**):Forest plots for the exposure of *IL-2ra*. **B** Forest plots for the exposure of *IP-10*. **C** Forest plots for the outcome of *IL-10*. **D** Forest plots for the outcome of *MIG*. **E** Forest plots for the outcome of* RANTES*

We explored the effects of 41 cytokines on MPN sequentially using a two-sample Mendelian randomization analysis (Supplementary Table 3). Only two cytokines (Interleukin-2 receptor, alpha subunit (*IL − 2rα*), and interferon gamma-induced protein 10 (*IP-10*) revealed suggestive associations with MPN risk after the Bonferroni correction. Genetically determined higher levels of circulating *IL-2rα* are suggestively positively associated with MPN risk [odds ratio (OR): 1.365,95% confidence interval (CI): 1.029–1.814, *P* = 0.032]. The other two complementary analytical methods Weighted median and MR Egger also obtained similar but not statistically significant results (Fig. 2A). Furthermore, Cochran's Q test did not reveal any heterogeneity (*P* = 0.268). A directional pleiotropy was also not discovered (MR egger-intercept = 0.021, *P* for MR egger-intercept = 0.710; *P* for MR PRESSO global test = 0.528) (Supplementary Table 5). Removing one SNP did not significantly change the results in the leave-one-out sensitivity analysis (Fig. 3A). Sensitivity analyses were conducted using the leave-one-out analyses to assess the reliability and stability of the results. The overall effect of the remaining instrumental variables was calculated by removing each SNP stepwise and observing whether the results changed after removing a single SNP. The results showed that removing individual SNPs did not significantly change the results of the exclusion sensitivity analyses (Fig. 3A). We did not find secondary phenotypes associated with SNPs that were used as instrumental variables on Pheno-Scanner's website.

We also observed a suggestive association between genetically determined higher circulating *IP-10* and a 55.3% increased risk of MPN (OR:1.553, 95% CI:1.088–2.216, *P* = 0.015), and it did not show heterogeneity (Cochrane Q test,* P* = 0.706)(Fig. 2A); nor did it show directional pleiotropy (MR egger-intercept = 0.085,* P* for MR egger-intercept = 0.305,* P* for MR PRESSO global test = 0.668) (Supplementary Table 5). Sensitivity analysis was conducted using leave-one-out studies, and the results showed that no individual study had any impact (Fig. 3B). We did not identify the SNP associated with other phenotypes on the Pheno-Scanner website, indicating that it does not increase the risk of MPN through the non-exposure pathway.

### Effect of MPN on inflammatory cytokines

In analyzing the effect of MPN on inflammatory factors, We found a suggestive association between genetically predicted MPN and levels of the cytokines *IL-10*, *MIG*, and *RANTES*. Genetically predicted MPN were suggestively associated with levels of interleukin-10 (*IL-10*) (BETA = 0.033,95% CI = 0.003 ~ 0.064, *P* = 0.032) and Monokine induced by interferon-gamma (*MIG*) (BETA = 0.052,95% CI = 0.002–0.102, *P* = 0.043) and Regulated on activation, normal T Cell expressed and secreted (*RANTES*) (BETA = 0.055,95% CI = 0.009 − 0.1, *P* = 0.018) using IVW methods. It is worth paying attention to the fact that in the *RANTES* analysis, although the MR-egger results were not statistically significant, the direction of the MR-egger results was inconsistent with the IVW results, suggesting that the *RANTES* results may be unreliable (Fig. 2B). In these findings, there was no indication of pleiotropy or heterogeneity. Supplementary Tables 4 and 6 provides a summary of the abovementioned findings. The leave-one-out analysis, meanwhile, revealed that all SNPs contributed to consistent causal estimates. (Fig. 3C-E). The analysis mentioned above demonstrated the validity of the results.

### The expression of *IP-10* and* IL-2ra* in various subtypes of MPN and their diagnostic value

**Fig. 4:**
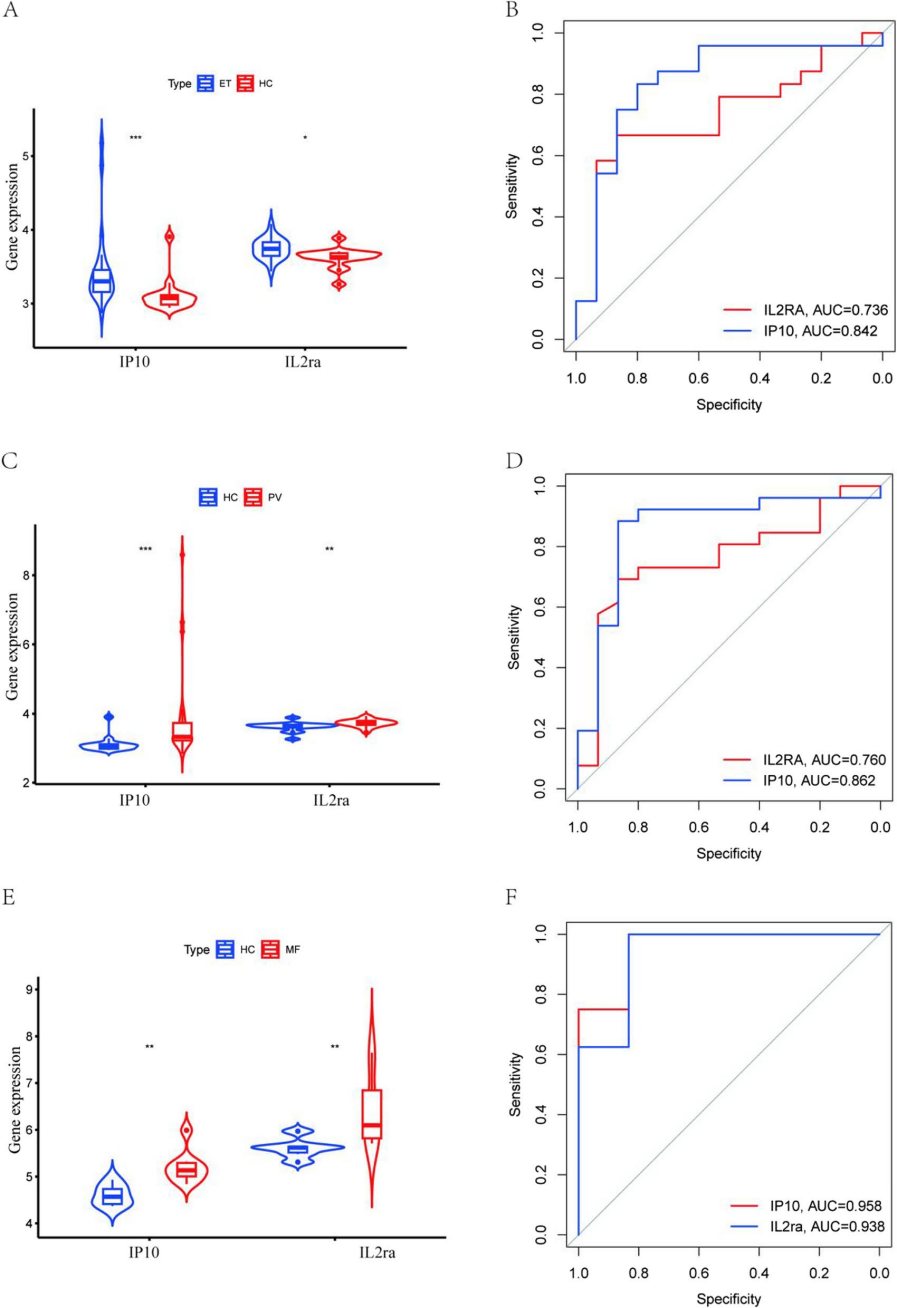
The GEO dataset analyses the expression of IP10 and IL2ra in each subtype of MPN and their diagnostic value.A: Expression of IP10 and IL2ra in ET and healthy donors; B: ROC curve of the prediction model based on IP10 and IL2ra to distinguish ET from healthy donors; C:IP10 and IL2ra expression in PV and healthy donors; D: ROC curve of the prediction model based on IP10 and IL2ra to distinguish PV from healthy donors; E:IP10 and IL2ra expression in MF and healthy donors; F: ROC curve of the prediction model based on IP10 and IL2ra to distinguish MF from healthy donors; ***P < 0.001, **P < 0.01,*P < 0.05,HC: Healthy donors

By analysing the effect of inflammatory cytokines on MPN, we found that higher levels of *IP-10*, *IL-2ra* were associated with increased risk of MPN. However, due to data limitations, we did not have the opportunity to further analyse the effect of *IP-10*, *IL-2ra* on the risk of developing each subtype of MPN using the MR approach. Therefore, in order to investigate the expression of *IP-10* and *IL-2ra* in each subtype of MPN and their diagnostic value, we analyzed their expression in healthy individuals and the three subtypes of MPN and their diagnostic value based on data from the GEO database, as shown in Fig. 4. Our results showed that *IP-10* and *IL-2ra* were elevated in ET, PV, and MF compared with normal subjects. The highest diagnostic efficacy for MF was based on the expression of *IP-10* and *IL-2ra* (IP10: AUC = 0.958, *IL-2ra*: AUC = 0.938); the areas under the ROC curves of *IP-10* and *IL-2ra* in ET, PV, and MF were greater than 0.7, suggesting that *IP-10* and *IL-2ra* may be used as potential diagnostic markers of MPN.

## Discussion

Using publicly available pooled data from GWAS, we conducted a bidirectional two-sample MR analysis of the potential causative relationship between inflammatory cytokines and MPNs, and our study supported a causal association between inflammatory cytokines and MPNs. We found suggestive evidence that levels of the genetically predicted circulating cytokines *IL-2rα*, and *IP-10* have a risk effect on MPNs. Reverse MR analysis found suggestive evidence of a positive causal effect of MPN on levels of the circulating cytokines *IL-10*, *MIG*, and *RANTES*. These findings passed sensitivity analyses and were not affected by heterogeneity or horizontal pleiotropy. To our knowledge, this investigation is anticipated to be the broadest and most thorough MR evaluation of links between genetically inflammatory cytokines and MPN risk to date.

The CXC chemokine family member interferon gamma-induced protein 10 (*IP-10*) is crucial for cell growth and proliferation [29].*IP-10* combines with the *CXCR3* receptor, being a key driver in cancer and autoimmune regulation [30]. Several observational studies have demonstrated the presence of aberrant *IP-10* expression in MPN patients, especially in PMF and PV, where *IP-10* expression is significantly elevated [31, 32]. Meanwhile, the serum level of *IP-10* was also correlated with the disease progression of MPN [32]. Our MR analysis suggests that elevated *IP-10* levels may contribute to MPN disease progression, which is consistent with results derived from observational studies. Previous basic research can explain our findings and the phenomena of observational studies in terms of pathogenesis.*IP-10* expression is reported to be required for the activation of the JAK signaling pathway [33]and its level correlates with *JAK2V617F* status [9, 34]. Therefore, *JAK* inhibition can reduce downstream chemokine *IP-10* production by disrupting T cell-induced macrophage activation [35]. However, stromal cells in the microenvironment can protect MPN clonal cells from *JAK2* inhibitors by secreting *IP-10*, which can promote disease progression. These discoveries underscore the importance of researching *IP-10* as a potential therapeutic target in the MPN tumor microenvironment and highlight the necessity of further studies on the exact mechanism of its role in MPN oncogenesis.

Our study also reveals a potential association between *IL2rα* and increased risk of MPN disease.*IL2rα* is an important component of *IL-2R*, a high-affinity receptor molecule highly expressed by activated T lymphocytes [36], and plays an important role in the regulation of T cell differentiation. Increasing *IL-2rα* expression on antigen-presenting cells (APCs) enhances the formation of memory T cells [37], and mutations in *IL-2rα* lead to decreased T cell function [38]. Accordingly, *IL-2rα* levels are associated with T-cell, B-cell, and immune system activation [36]. It has been demonstrated that conditions linked to cellular immune activation correlate with increased *IL-2rα* [39, 40]. Additionally, a few observational studies have shown a connection between *IL2r* and MPN. Katerina et al. found that serum levels of *IL-2rα* were significantly elevated in patients with MPN compared to normal individuals [14], which was confirmed by further studies, where *IL2rα* levels were correlated with overall survival in patients with MF in MPN [41], and levels of *IL-2ra* in patients with MPN were positively correlated with disease progression and bone marrow angiogenesis [42]. According to the findings of our MR investigation, elevated levels of *IL-2rα* in the circulatory system may accelerate the development of MPN disease. This finding is not only consistent with the results of observational studies but also compensates for the shortcomings of small sample sizes and potential confounders in the observational studies mentioned above and provides more reliable evidence for the association between *IL2rα* and MPN at the level of genetics, emphasizing the importance and necessity of further investigating the role of*IL2rα* in the development of MPN disease.

Both our analyses and previous studies suggest that *IP-10* and *IL2rα* may play an important role in MPN disease development. Considering the heterogeneity of the three subtypes of MPN, we sought to explore the effects of *IP-10* and *IL2rα* on the disease risk of each subtype of MPN. Unfortunately, due to the limitation of the dataset, we had no way to further explore the relationship between *IP-10* and *IL2rα* and different subtypes of MPN. Therefore, we initially analyzed the expression and diagnostic value of *IP-10* and *IL2rα* in each subtype of MPN using the GEO database. We were surprised to find that *IP-10* and *IL2rα* not only had elevated expression in the three subtypes of MPN compared to healthy individuals, but also had the potential to serve as independent biomarkers. This is consistent with our MR analysis that high expression of *IP-10*, *IL2rα* increases the risk of MPN disease. This greatly encourages our confidence in further exploring the role of *IL2rα*, *IP-10* in MPN at a later stage.

Positive MR analysis has revealed the role of inflammatory cytokines, particularly *IP-10* and *IL-2rα*, in MPN disease progression. Indeed, MPN cells can also release large amounts of pro-inflammatory products, which in turn cause genomic instability and drive clonal myeloproliferation [43, 44].To explore the effect of MPN disease on inflammatory cytokines, we performed a reverse MR analysis. The inverse MR analysis showed a potential positive correlation between genetically predicted MPN and the levels of cytokines *IL-10*, *MIG*, and *RANTES*, and that MPN could promote slightly increased levels of the above cytokines. Inverse MR analysis revealed a potential positive correlation between genetically predicted MPN and levels of the cytokines *IL-10*, *MIG*, and *RANTES*, with MPN promoting slightly elevated levels of the aforementioned cytokines, which is consistent with observational findings [9, 45–47]. Our review of the literature revealed that aberrantly expressed *IL-10*, *MIG*, and *RANTES* are all associated with premature atherosclerosis, a devastating consequence of chronic inflammation in the MPN [48–50].MIG binds to the receptor *CXCR3* and not only participates in the recruitment of T cells to peripheral sites of inflammation [51]but also chemotactically recruits monocytes/macrophages to sites of inflammation. Activated inflammatory cells release pro-inflammatory factors to induce an inflammatory response [52], which promotes atherosclerosis. *RANTES* is one of the chemokines highly expressed upon platelet activation, and *RANTES* released by activated platelets facilitates the formation of atherosclerotic lesions by platelet-monocyte aggregation [53, 54], and *RANTES* also regulates local inflammatory processes and atherosclerosis progression by mediating CD4 + T-cell homing [55]. It is interesting to note that *IL-10* appears to be a protective factor against atherosclerosis, a common clinical symptom of MPN, and that, as an anti-inflammatory cytokine, *IL-10* can attenuate atherosclerotic lesions by preventing dilation of inflamed areas, decreasing the size of plaques, and other mechanisms [56]. Specifically, *IL-10* attenuates atherosclerotic lesions by inhibiting macrophage activation, as well as inhibiting the expression of matrix metalloproteinases, proinflammatory cytokines, and cyclooxygenase-2 in lipid-loaded and activated macrophage foam cells [57, 58]. Therefore, it is necessary to investigate the correlation and mechanism between the elevated circulating levels of MPN-promoting inflammatory cytokines *IL-10*, *MIG*, and *RANTES* and the common clinical complications of MPN, and to provide the possibility of targeting the above cytokines to alleviate the clinical complications.

Our study has several advantages. (1) The link between inflammatory cytokines and MPN risk is explained for the first time in magnetic resonance research. (2) Unlike observational studies, our study minimized confounders and reverse causality, providing a reliable causal relationship between MPN and inflammatory cytokines. (3) Our research data were sourced from the openly available GWAS database, which houses a significant volume of original research data, and thus gives this study a solid guarantee.

There are also some limitations to our study. In the first place, all participants in the dataset we used were of European ethnicity, which limits our ability to generalise our findings to other ethnicities. It is well known that Mendelian randomisation investigates the effect of genotypic variation (exposure) on phenotype (outcome) from a genetic perspective. Bias caused by confounding variables or reverse causality is avoided [18].In reality, however, it is well known that the level of gene expression determines the unique characteristics of a cell, that differences in disease prevalence between populations are associated with the frequency of alleles that regulate polymorphisms, and that differences in allele frequencies between racial groups have highly significant phenotypic consequences [59].Significant differences in gene expression phenotypes have been reported for at least 25 percent of genes between Europeans and Asians, and specific genetic variants (allele frequencies) between populations are the main cause of these differences [59].Therefore, in Mendelian randomisation analyses, genetic differences in quantitative phenotypes between different ethnic groups may be functionally equally important. Environmental, genetic, dietary, and lifestyle factors in different racial groups may influence phenotypic results [60], so we think it is unavoidable that the results of MR may differ between races due to residual confounding and selection bias [61, 62].This is our limitation in this study. Therefore, whether elevated *IP-10* and *IL-2ra* increase the risk of MPN prevalence in other populations requires specific analyses of gene expression variation for particular populations. Future studies will also need to enhance the analysis of gene expression variation between populations to improve understanding of the underlying genetics and population differences observed in complex genetic diseases. In the second, there are three subtypes of MPN, and due to the limitations of the GWAS dataset, we have not specifically stratified to explore the relationship between cytokines and the different subtypes of MPN. Finally, after Bonferroni correction, no cytokines showed statistically significant associations with MPN risk, and only two of them (IP-10, IL-2rα) showed suggestive associations.

In conclusion, our study suggests that elevated circulating levels of *IP-10* and *IL-2rα* are associated with a high risk of MPN. Potential positive correlation between genetically predicted MPN and levels of the cytokines *IL-10*, *MIG*, and *RANTES*. Our results show that cytokines play a significant role in the pathophysiology of MPN. Further research is required on the potential use of these biomarkers for the prevention and treatment of MPN.

### Supplementary Information

**Supplementary Material 1**.

**Supplementary Material 2**.

**Supplementary Material 3**.

**Supplementary Material 4**.

**Supplementary Material 5**.

**Supplementary Material 6**.

## Data Availability

The datasets analyzed during the current study are available in the FinnGen repository, More details in FinnGen are described at https://r9.finngen.fi.
